# Martial arts as a mental health intervention for children? Evidence from the ECLS-K

**DOI:** 10.1186/1753-2000-3-32

**Published:** 2009-10-14

**Authors:** Joseph M Strayhorn, Jillian C Strayhorn

**Affiliations:** 1 Drexel University College of Medicine, Department of Psychiatry, 2900 W. Queen Lane, Philadelphia, PA 19129, USA; 2 University of Pittsburgh School of Medicine, Department of Psychiatry, 3811 O'Hara Street, Pittsburgh, PA 15213, USA; 3 3263 Seasons Drive, Wexford, Pennsylvania 15090, USA

## Abstract

**Background:**

Martial arts studios for children market their services as providing mental health outcomes such as self-esteem, self-confidence, concentration, and self-discipline. It appears that many parents enroll their children in martial arts in hopes of obtaining such outcomes. The current study used the data from the Early Childhood Longitudinal Study, Kindergarten class of 1998-1999, to assess the effects of martial arts upon such outcomes as rated by classroom teachers.

**Methods:**

The Early Childhood Longitudinal Study used a multistage probability sampling design to gather a sample representative of U.S. children attending kindergarten beginning 1998. We made use of data collected in the kindergarten, 3^rd ^grade, and 5^th ^grade years. Classroom behavior was measured by a rating scale completed by teachers; participation in martial arts was assessed as part of a parent interview. The four possible combinations of participation and nonparticipation in martial arts at time 1 and time 2 for each analysis were coded into three dichotomous variables; the set of three variables constituted the measure of participation studied through regression. Multiple regression was used to estimate the association between martial arts participation and change in classroom behavior from one measurement occasion to the next. The change from kindergarten to third grade was studied as a function of martial arts participation, and the analysis was replicated studying behavior change from third grade to fifth grade. Cohen's f^2 ^effect sizes were derived from these regressions.

**Results:**

The martial arts variable failed to show a statistically significant effect on behavior, in either of the regression analyses; in fact, the f^2 ^effect size for martial arts was 0.000 for both analyses. The 95% confidence intervals for regression coefficients for martial arts variables have upper and lower bounds that are all close to zero. The analyses not only fail to reject the null hypothesis, but also render unlikely a population effect size that differs greatly from zero.

**Conclusion:**

The data from the ECLS-K fail to support enrolling children in martial arts to improve mental health outcomes as measured by classroom teachers.

## Background

The impetus for this study came when the first author, a child and adolescent psychiatrist, noticed that a significant fraction of patients in his practice were enrolled in martial arts training. None of the parents of these children sought better fighting skills for their children, but rather mental health outcomes such as discipline, confidence, or concentration skills. This observation led the authors to wonder how martial arts training affects mental health outcomes, and to seek a larger and more representative sample with which to study this question.

The marketing materials from martial arts studios are directly aimed toward the attainment of positive mental health outcomes. We conducted a quick informal sampling of studio marketing materials by entering into Google's search engine the words "martial arts children" and selecting the first 15 websites that appeared for children's martial arts studios. Pitches for the improved "self-esteem," "self-image," or "self-assurance" occurred in 60%; improved "focus," in 67%; more "confidence" or "self-confidence," in 80%, and "discipline," "self-discipline," "self-control," or "self-direction," in 100%. One of these studios, in a not-atypical web advertisement, listed as outcomes of martial arts training not only all four of the above, but also anger management, study skills, respect for others, problem-solving, help with ADHD, and nonviolent conflict resolution [1]. These goals overlap highly with those of mental health services for children.

Indeed, if the marketing claims were completely valid, martial arts studios would be a logical alternative to a great portion of the child and adolescent mental health system. If there is even a good chance that these claims are true, the mental health research field has an obligation to investigate them thoroughly, by, for example, conducting head-to-head clinical trials of psychotherapy versus martial arts. And clinicians should be able to give informed answers to parents' questions about the mental health benefits of such training.

At least two empirical studies support the concept of martial arts as a mental health intervention. A randomized study of Tae Kwon Do versus traditional physical education in 207 elementary school students revealed greater improvement in the martial arts group in several variables, including prosocial behavior, classroom conduct, and performance in mental math [2]. Another martial arts versus waitlist control comparison conducted in a middle school found that martial arts students improved over baseline on twelve behavioral variables, whereas controls improved on five and deteriorated on eight, including teacher rated violence [3].

On the other hand, when aggressiveness is one of the major dimensions of childhood psychopathology, there is something counterintuitive about improving mental health by teaching fighting skills. Despite the emphasis some martial arts teachers give to nonviolence, much of the subject matter of martial arts is proficiency in violence: punching and kicking maneuvers that can harm or kill people. Competitive martial arts can be a violent sport. For example, a videotape analysis of a 2001 Tae Kwon Do tournament in South Korea concluded that the frequency of head blows and concussions was high: there were nearly half as many head blows recorded as competitors in the tournament [4]. Even if students are not permitted to hurt one another, the learning theory concept of "response class" would predict that highly repetitive practice of delivering blows to another human being would tend to decrease inhibitions about aggressive acts and increase the likelihood of aggressive behavior.

Large longitudinal studies are capable of looking at a representative sample of martial arts instruction and a representative sample of students. One such longitudinal study came to just the opposite conclusions from the experiments mentioned above: power sports (including boxing and wrestling, as well as martial arts) were associated with elevated levels of violent behavior as well as nonviolent antisocial behavior outside sports [5].

Other studies raise further questions about the nonviolence and harmony supposedly promoted by martial arts. A study of adolescent mass murderers found that preoccupation with violent themes, of which martial arts was a frequent example, was documented in about half of the sample of murderers [6]. A laboratory experiment found that allowing children to watch videos of martial arts (a condensed version of *The Karate Kid*) versus Olympic competition scenes seemed to desensitize children to real life aggression in that, after observing the martial arts, it took them longer to decide to get help when two other children were portrayed as starting to fight [7]. A study that followed children in martial arts for two years concluded that karate training had neither positive nor negative effects on aggressiveness scores, while judo increased anger [8].

Much of the research on the effects of martial arts has involved convenience samples, a single martial arts intervention tailored for the study, and fairly small sample sizes. The present study uses the database of The Early Childhood Longitudinal Study, Kindergarten Class of 1998-1999 (ECLS-K). This study obtained a large sample designed to be representative of U.S. children; the cohort has been followed over several years.

There are several major advantages to using this data set to approach this question. The sample size is large. The martial arts training received by students in this sample does not represent a particular intervention designed for the study, but something of an average of martial arts as it is delivered in the United States as a whole. It is likely that most of the teachers who rated children's behavior in the ECLS were blind to the students' having received martial arts training. When intervention is carried out in one school, the creation of a group climate in martial arts class means that the students are probably not independently functioning "units of analysis," but affect one another; this is not the case in a large longitudinal survey. Finally, the longitudinal design allows us to look at change over time, and thus come closer to drawing causal inferences than would be possible with cross-sectional correlations.

The present study examined, as the outcome variable, teachers' ratings of children's behavior. The null hypothesis was that participation in martial arts has no effect upon the change in classroom behavior from one measurement period to the next.

## Methods

### Participants

The ECLS-K study enrolled a total of 21,260 children from 1,277 kindergarten classrooms of public, Catholic, and non-Catholic private schools across the United States. Informed consent was obtained from each family. The sample was increased by 165 children in first grade in order that children who attended first grade without attending kindergarten would also be represented. The study was funded by the U.S. Department of Education and conducted under the auspices of the National Center for Education Statistics. The data from this study are available to the public. Through multistage probability sampling, the ECLS-K study strived for generalizability to the population of U.S. children eligible for kindergarten in 1998. The multistage sample was orchestrated in the following way: "In the base year the primary sampling units (PSUs) were geographic areas consisting of counties or groups of counties. The second stage units were schools within sampled PSUs. The third and final stage units were students within schools." (p. 4-1) [9]

Despite the major goal of attaining a nationally representative sample, because of incomplete responses, incomplete data, and occasional deliberate oversampling of certain segments of the population, the unweighted cases in various parts of the data set still do not constitute a representative sample of the population as a whole. As the sample sizes reported in our study demonstrate, the effort to conduct personal interviews with parents several times over a six year interval results in a great deal of missing data. In order to correct for the above mentioned factors, weights were assigned to cases by the National Center for Educational Statistics, using a strategy designed to ensure that weighted subsamples would be as nationally representative as possible. These weights are numbers assigned to each case in the data set that are have the effect of allowing certain cases to contribute more and other cases less to the overall statistics obtained. Table 1 presents the ethnicity characteristics of the K-3 sample, weighted and unweighted. Both weighted and unweighted samples comprised very close to 50% males and 50% females.

**Table 1:** Ethnicity Distribution, grade K-3 Sample

**Race**	**Weighted**	**Unweighted**
White, not Hispanic	66.8%	66.2%
Black	11.5%	10%
Hispanic, race specified	7.5%	7.0%
Hispanic, race not specified	8.1%	7.0%
Asian	2.0%	4.5%
Hawaiian or Pacific Islander	0.6%	1.3%
American Indian or Alaska native	1.5%	1.5%
More than one race	2.1%	2.5%

Ordinary statistical analyses make the assumption of a simple random sample from a population, which results in observations that are independent of one another. The multistage sampling design creates a violation of the assumption of independence because, for example, students in the same school are probably more alike one another than students in different schools.

In order to deal with the complexities of the multistage sampling design and the weighting procedures, specialized statistical software, the AM software package [10], has been designed specifically for studies of this sort. Such software enables two methods of estimation of standard errors in statistical analyses that give more accurate estimates than traditional methods would allow. The method used for this study is the jackknife technique, which falls into the category of resampling statistical methods [11]. Resampling statistical techniques rely upon selecting multiple subsamples from the initial sample and using such data to estimate the variability in the population more accurately than by assuming a certain distribution.

We used two main panel weights for the purposes of the six main analyses in this article: C245CW0 and C56CW0. The first is used when analyses involve children at the kindergarten and 3^rd ^grade stage, and the second is used when making analyses of children at the 3^rd ^and 5^th ^grades. For each of these analyses, the NCES provided ninety replicate weights to be used by the software to create ninety subsamples; the values of given statistics for all these subsamples are entered into calculations estimating standard errors for the statistics in question.

In this study we examined the effect of martial arts upon change in classroom behavior from kindergarten to third grade. We replicated this analysis with the change in behavior from third to fifth grade. For each of these two analyses we used the cases available in the ECLS for that particular analysis. Thus the sample sizes, and the samples, differ between the two analyses.

### Measures

Participation in martial arts was measured through several questions in a structured, face to face, individual interview conducted by research staff with parents of children in the project. First, the parents were presented with this question: "In the last 12 months, did {CHILD} regularly get exercise through any of the following organizations?" Those who indicated that their children had gotten exercise then answered a following question: "What types of exercise or physical activity did {CHILD} get at the places you just mentioned?" Then martial arts were specifically inquired about, and the parent answered yes or no.

The measure of classroom behavior was a composite of questions on a questionnaire, the Social Rating Scale, which was derived from the Social Skills Rating System [12] and completed by teachers during each wave of the study. Four questions constituted a self control subscale: respecting the property rights of others, controlling temper, accepting peer ideas for group activities, and responding appropriately to pressure from peers. Six involved "approaches to learning": attentiveness, task persistence, eagerness to learn, learning independence, flexibility, and organization. Five involved interpersonal skills: forming and maintaining friendships; getting along with people who are different; comforting or helping other children; expressing feelings, ideas, and opinions in positive ways; and showing sensitivity to the feelings of others. Five involved externalizing problem behaviors: the frequency with which a child argues, fights, gets angry, acts impulsively, and disturbs ongoing activities. Four involved internalizing problem behaviors: the apparent presence of anxiety, loneliness, low self-esteem, and sadness. For the third and fifth grade administrations of this questionnaire, two items were added: an approaches to learning item, "child follows classroom rules," and an externalizing item asking about the frequency with which a child talks during quiet study time. Although there were only four items in the official self-control subscale, the entire scale is weighted very heavily toward items involving self-control. There is hardly an item on the teacher questionnaire that does not involve, in some way, the dimensions of self-control and self-confidence that many parents seek as outcomes when their children study martial arts. The items on this questionnaire highly resemble those of other questionnaires used by mental health professionals to assess children's psychological health.

Each question was rated on a 1 (never) to 4 (very often) Likert scale. Negative items' scores were subtracted from five, so that each item had a range where 1 was worst and 4 was best. The average of the twenty-four items for kindergartners and the twenty-six items for third and fifth graders was used for our analyses. The five subscales were each internally consistent at the outset: the split half reliabilities for the various subscales of the teacher ratings were high. For fifth grade, for example, the split half reliabilities ranged from .77 for internalizing to .91 for approaches to learning [9]. Furthermore, the correlations between these various subscales were reasonably high. In fifth grade, for example, the unweighted correlations ranged from .31 to .81; the average correlation was .56. These correlations appeared high enough to justify combining the items into one scale, measuring the favorableness of each child's classroom behavior.

In order to check the internal consistency of the composite classroom behavior rating, we computed the coefficient alpha for this scale, counting the five subscales as five "items" which the composite scale comprised.

### Data Analyses

The data were extracted into SPSS statistical software [13], where creation of composite variables, recoding of missing values, merging of data files, checking of coefficient alphas, and checking for interaction effects were accomplished. Thence the data were exported to AM software for the major analyses, which made use of the weights and replicate weights and took into account the multistage sampling.

When studying the change in classroom behavior between time 1 and time 2, how should the martial arts variable be coded? Cohen and Cohen [14] summarize the answer to this question: "The idea of dummy-variable coding is to render the information of membership in one of g groups by a series of g-1 dichotomies." In the present case, the martial arts variable with respect to kindergarten and third grade comprises membership in four groups: 1) a group of children who participated in martial arts in kindergarten but not third grade, 2) a group who participated in third grade but not kindergarten, 3) a group who participated both at kindergarten and third grade, and 4) a group who participated at neither time period. Membership in these four groups was coded by three dichotomous variables. The "K, not 3" variable was coded 1 for children in the first group and 0 for all others; the "3, not K" variable was coded 1 for children in the second group and 0 for all others; the "BothK3" variable was coded 1 for children in the third group and 0 for all others. The fourth group, who participated in martial arts at neither time, does not need another variable to be defined, because the value of 0 for each of the three other variables specifies this group. Table 2 summarizes the dummy variable coding for the K to 3 sample:

**Table 2:** Dummy Variable Coding for K-3 Sample. Four groups are coded by three variables, as follows:

	**K, not 3 code**	**3, not K code**	**BothK3 code**
Martial in K, not 3	1	0	0

Martial in 3, not K	0	1	0

Martial in K and 3	0	0	1

Martial in neither K nor 3	0	0	0

Thus these three variables completely represent the four possible ways that children can assort themselves into groups by participation in martial arts at the two time points.

In the multiple regression that is the definitive test of whether martial arts influences change in classroom behavior, the time two behavior is the dependent variable. The time one behavior is entered as the first independent variable. Then the entire set of three dichotomies is entered as the second block. If the whole block of three variables representing martial arts participation does not significantly increase the explained variance over that explained by the time one behavior alone, we fail to reject the null hypothesis.

In addition, the regression coefficients for these three dummy variables are individually meaningful, in terms of various time courses that the effect of martial arts could take. To the extent that martial arts participation in the time one year sets off a gradual change manifesting itself finally in improvement evident by the two year, regardless of time two participation, K, not 3 would have a larger coefficient. If the effect of martial arts is more short term, with classroom behavior responding quickly while martial arts participation is going on, this sort of effect would increase the 3, not K regression coefficient. If martial arts participation had an effect on behavior that was cumulative, but which depended upon continued participation, the BothK3 coefficient would be raised. If all three of the regression coefficients are close to zero, then no combination of martial arts participation at either of the two measurement periods shows evidence of any effect.

Exactly the same sort of dummy-variable coding applies to the study of change in behavior between third and fifth grade.

For the regressions used in this study, the jackknife technique was used to estimate standard errors. There was a regression analysis for each of the two time periods (i.e. kindergarten to 3^rd ^grade and 3^rd ^grade to 5^th ^grade). We computed confidence intervals for the regression coefficients corresponding to the three martial arts variables.

Each of the regression analyses that we report represents a separate test of the effect of martial arts training on classroom behavior. Each of these tests could have been a separate piece of research; it so happened that both analyses were available from this data set. Accordingly, there are two different samples reported upon in this article, comprising the cases which had complete data for the measurement periods in question. To have restricted the sample to only those cases available for both analyses would have unnecessarily discarded useful data. We also did not use imputation methods for missing data, because the weighting system established by the NCES was meant to accomplish the representativeness of samples that would have been the goal of imputation.

For both regressions we did, we calculated an effect size for martial arts, using Cohen's f^2^, which is equal to (R^2^_AB _- R^2^_A_)/(1-R^2^_AB_), where R^2^_A _in this case is the variance in time two behavior accounted for by the time one behavior rating only (that is, the bivariate regression of time two behavior on time one behavior) and R^2^_AB _is the variance accounted for by the multiple regression model including both time one behavior and the three dichotomies representing martial arts. Thus, this effect size measures the improvement in prediction of time two behavior that is attained by adding martial arts to the regression model originally consisting only of time one behavior as the independent variable. By convention, f^2 ^values of .02, .15, and .35 are called small, medium, and large effect sizes respectively [15].

## Results

### Participation in martial arts

What fractions of students in our two samples participated in martial arts? For the kindergarten through 3^rd ^grade sample, the percents of children participating were 3.3% for kindergartners and 7.5% for third graders. For the third to fifth grade sample, the percents participating were 7.2% for third graders and 7.1% for fifth graders.

Of those who were in martial arts in one time period, how many continued to the next time period? The answer is that, of the children who participated in kindergarten, 27.4% remained in participation in 3^rd ^grade. Of the children who participated in 3^rd ^grade, 6.8% participated in 5^th ^grade. Thus among these children there appears to be a fairly high turnover in participation in martial arts as opposed to stable participation over years.

### Checks on the characteristics of measures

Each of the distributions for the behavior ratings was approximately mound-shaped and symmetrical, a little skewed to the left, but roughly consistent with a normal distribution. Normal probability plots also did not deviate much from what is expected of a normal distribution.

The (unweighted) coefficient alphas for the behavior ratings in each of the three measurement periods were computed, treating each of the five subscales as one item. The coefficient alphas were 0.86 for the kindergarten administration, and 0.87 for both the third grade and fifth grade administrations. These internal consistencies are more than adequate to justify combining subscales into single composite scales [16] (p.245). Combining the five subscales into one composite can be expected, given the Spearman-Brown formula, to result in a more reliable measure of behavior than any one subscale by itself [16] (p 243).

An important requisite for meaningful regression analyses and analyses of the effects of martial arts is that the outcome variables possess a sufficient degree of stability from one year to the next. The correlation between kindergarten behavior variable and the third grade behavior variable, was 0.52 (n = 8,851). The correlation between the third grade behavior variable and the fifth grade behavior variable was 0.59 (n = 4,896). Given that the observers were different and that the children were two to three years older than they were for the previous measurement, these correlations give evidence for adequate reliability for the behavior ratings.

### Association of martial arts with change in behavior

Tables 3 and 4 present the means and standard deviations for behavior at each of the times sampled. These permit a direct inspection of the changes over time for the two groups.

**Table 3:** Means and standard deviations of outcome variables, by martial arts group, kindergarten to third grade sample (n = 8851)

**Group**	**Grade K Behavior:** **mean (sd)**	**Grade 3 Behavior** **mean (sd)**	**N for Group**
Martial arts in K, not 3	3.28(0.46)	3.16 (0.57)	212

Martial arts in 3, not K	3.15 (0.51)	3.12 (0.52)	584

Martial arts both K and 3	3.25 (0.51)	3.17 (0.52)	80

Martial arts Neither	3.26 (0.50)	3.19 (0.52)	7975

**Table 4:** Means and standard deviations of outcome variables, by martial arts group, third to fifth grade sample (n = 4,896)

**Group**	**Grade 3 Behavior:** **mean (sd)**	**Grade 5 Behavior** **mean (sd)**	**N for Group**
Martial arts in 3, not 5	3.07 (0.52)	3.12 (0.53)	328

Martial arts in 5, not 3	3.03 (0.58)	3.11 (0.58)	323

Martial arts both 3 and 5	3.00 (0.65)	3.14 (0.38)	24

Martial arts Neither	3.15 (0.53)	3.18 (0.51)	4221

Table 5 presents the results of the regression for the kindergarten to third grade study, and table 6 presents the results for the third to fifth grade study.

**Table 5:** Regression results: dependent is classroom behavior, grade 3 (n = 8851)

**Independent variable**	**Regression Coefficient**	**SE of regression coefficient**	**95% Confidence Interval for regression coefficient**	**p-value for regression coefficient**
Classroom Behavior, KG	0.535	0.018	0.500 to 0.570	<0.0005
K, Not 3 Martial Arts	-0.043	0.047	-0.135 to 0.049	0.365
3, Not K Martial Arts	-0.018	0.030	-0.077 to 0.041	0.538
Both K, 3 Martial Arts	-0.014	0.055	-0.122 to 0.094	0.802

**Table 6:** Regression results: dependent is classroom behavior, grade 5 (n = 4896)

**Independent variable**	**Regression Coefficient**	**SE of regression coefficient**	**95% Confidence Interval for regression coefficient**	**p-value for regression coefficient**
Classroom Behavior, Grade 3	0.566	0.022	0.523 to 0.610	<0.0005
3, Not 5 Martial Arts	-0.014	0.035	-0.083 to 0.055	0.685
5, Not 3 Martial Arts	-0.004	0.049	-0.100 to 0.092	0.938
Both 3, 5 Martial Arts	0.051	0.078	-0.102 to 0.204	0.516

The results of both these regression analyses are consistent: the martial arts variable (represented by the set of three dummy variables) is not statistically significant and does not improve the prediction achieved by time one behavior alone. For the K to 3 analysis, kindergarten behavior accounted for 26.9% of the variance in third grade behavior; addition of the set of martial arts variables did not change the R^2 ^at all. Likewise, for the 3 to 5 analysis, third grade behavior accounted for 35.2% of the variance in fifth grade behavior, and the regression model with the three martial arts variables added resulted in an identical R^2^. The Cohen's f^2 ^statistic was 0.000 for the martial arts variable for both analyses.

Furthermore, 95% confidence intervals for the regression coefficients for the martial arts variables for both analyses encompassed zero and had both upper and lower bounds close to zero. With behavior measured on a scale of 1 to 4 (the average of all of the items), the largest deviation from zero for either the upper or lower bound of a confidence interval was about a fifth of a point. The results allow us to infer an effect size very close to zero.

### Check of assumption of non-interaction

One of the assumptions for the use of the multiple regression to test the effects of the martial arts variable is the lack of an important interaction between martial arts and the prescore for the outcome variable in question. For both regressions we checked the interaction assumptions by using unweighted regressions, and adding to the model three interaction terms, which are the product of the three martial arts dichotomies and the prescore for classroom behavior. The interaction effect is measured by the significance and R^2 ^change for the block of 3 interaction terms when they are added to the model. For the kindergarten to third grade analysis, this interaction was not significant (p = 0.48), R^2 ^change = 0.000. For the third to fifth grade analysis, the interaction term was significant with p = .038. However, when using samples sizes as large as in this study, a statistically significant interaction term does not necessarily mean a practically significant interaction. The R^2 ^change was 0.001, and the Cohen's f^2 ^effect size for this interaction was calculated to be .0014, which is well below the lower limit for a small effect size. Our conclusion from these analyses is that the slopes of the regression lines for the martial arts group and the non-martial arts group are close enough to identical that the regression models we report do not violate assumptions.

### Check of robustness of the findings if the weights are ignored

We could not help wondering how strongly the results of this research depended upon the accuracy with which the panel weights and replication weights were assigned to the cases in this study, and the accuracy with which the specialized software was created. For this reason we checked to see how the results would have differed if the complexities of panel weights, replication weights, the jackknife method, special software, and the efforts to obtain a representative weighted sample were simply ignored. In other words, what if the samples we dealt with were treated simply as convenience samples, without any efforts to correct for non-representativeness? When we redid the analyses using ordinary least-squares regressions using SPSS, the p-values for the regression coefficients were slightly different, but all still nonsignificant. The f^2 ^effect sizes were nearly identical: for the kindergarten to third grade analysis, the effect size was 0.000 and for the third to fifth grade analysis, the effect size was 0.001. Thus, with no reliance on corrections meant to achieve greater statistical validity, the conclusions would have been identical.

## Discussion

It is a statistical maxim that one can never prove the null hypothesis, but only fail to reject it. And indeed, it is hard to imagine that any training experience people undergo would produce an effect on behavior of exactly 0, when carried to an infinite number of decimal places. However, with large sample sizes, confidence intervals with bounds close enough to zero can lead us to comfortable conclusions that population effect sizes larger than trivial ones are improbable. The current study is a case in point.

An important limitation of this study is that the ECLS-K gathered only one bit of information on the child's martial arts participation at any given measurement occasion. A study designed specifically to assess the effect of martial arts would have gathered data on the start and end dates and frequency of training, and the specific curricula of the various studios. It is conceivable that we failed to find effects because too few students persisted at the study of martial arts long enough. We would be very curious to know the average length of training.

However, even with the length and specific type of training unknown, we can regard the length and type of training as representative of the "average" length and type obtained in the U.S. It does seem to us that if martial arts were, on the average, as effective an intervention as its proponents believe, participation as measured by the simple answer to whether the child is participating in martial arts would have revealed at least a tiny visible effect, given the more than adequate sample size and given the reliability of the behavior rating variable.

The claims of martial arts studios and the expectations of many parents that martial arts will improve self-control and self-confidence contrast with the near-zero effect sizes found in these analyses. Changing students' behavior outside the classroom in a way that generalizes to the classroom is, we suspect, in general not an easy task. This study fails to find evidence that martial arts training achieves this goal.

It's important to remind ourselves that educational interventions such as martial arts are not homogeneous. Martial arts as taught by one practitioner may be totally different from that taught by another. One practitioner may emphasize self-control and emotional regulation, whereas another might emphasize self-defense or preparation for competition, and a third might actually promote aggression; the intervention can be very different depending on who is teaching it. Thus it is possible that the close-to-zero effects that we report here are an average of positive and negative effects. Thus our results do not rule out the possibility that some studios regularly achieve positive effects, and others achieve negative ones. It could also be that even within individual studios, there are net positive effects on some children from encouraging self-discipline and respect, which are cancelled by net negative effects on others from practice of physical aggression. The current study probably offers a reasonable estimate of the effect on classroom behavior of enrolling an elementary school child in "the average" U.S. martial arts studio; and continuing training an "average" length of time. The estimate for such training is a zero effect.

## Conclusion

The strategy of enrolling elementary school aged children in martial arts training in order to improve self-control, self-confidence, concentration, and other mental health outcomes, as measured in classrooms by teachers, is not supported by the data of this study. There may be other reasons for enrolling children in martial arts, for example physical fitness or self-defense, or effects on behaviors outside the school classroom; these are beyond the scope of this study.

## Competing interests

The authors declare that they have no competing interests, other than the practice of clinical child and adolescent psychiatry by the first author.

## Authors' contributions

JMS conceived the topic and obtained the ECLS-K data set. The authors together searched and read and summarized preexisting literature on this topic. Both authors spent several hours studying and discussing the documentation furnished with the data set. The authors together deliberated and decided on the nature of the statistical analyses. JCS used the statistical software packages to actually carry out the analyses, with the consultation of JMS. The authors together constructed the tables, prepared the references, and wrote and revised the manuscript. Both authors approved the final version.
